# Drug repositioning in thyroid cancer treatment: the intriguing case of anti-diabetic drugs

**DOI:** 10.3389/fphar.2023.1303844

**Published:** 2023-12-11

**Authors:** Alessia Greco, Francesca Coperchini, Laura Croce, Flavia Magri, Marsida Teliti, Mario Rotondi

**Affiliations:** ^1^ Department of Internal Medicine and Therapeutics, University of Pavia, Pavia, Italy; ^2^ Laboratory for Endocrine Disruptors, Unit of Endocrinology and Metabolism, Istituti Clinici Scientifici Maugeri IRCCS, Pavia, Italy

**Keywords:** drug-repositioning, thyroid-cancer, anti-diabetic, metformin, diabetes

## Abstract

Cancer represents the main cause of death worldwide. Thyroid cancer (TC) shows an overall good rate of survival, however there is a percentage of patients that do not respond or are refractory to common therapies. Thus new therapeutics strategies are required. In the past decade, drug repositioning become very important in the field of cancer therapy. This approach shows several advantages including the saving of: i) time, ii) costs, iii) *de novo* studies regarding the safety (just characterized) of a drug. Regarding TC, few studies considered the potential repositioning of drugs. On the other hand, certain anti-diabetic drugs, were the focus of interesting studies on TC therapy, in view of the fact that they exhibited potential anti-tumor effects. Among these anti-diabetic compounds, not all were judjed as appropriate for repositioning, in view of well documented side effects. However, just to give few examples biguanides, DPP-4-inhibitors and Thiazolidinediones were found to exert strong anti-cancer effects in TC. Indeed, their effects spaced from induction of citotoxicity and inhibition of metastatic spread, to induction of de-differentiation of TC cells and modulation of TC microenvironment. Thus, the multifacial anti-cancer effect of these compounds would make the basis also for combinatory strategies. The present review is aimed at discuss data from studies regarding the anti-cancer effects of several anti-diabetic drugs recently showed in TC in view of their potential repositioning. Specific examples of anti-diabetic repositionable drugs for TC treatment will also be provided.

## Introduction

Cancer mortality represents one of the most significant social, medical, and scientific primary challenges. The course of history teaches us that several effective anti-cancer drugs have been developed from ancestral compounds. The first employed anti-cancer drugs were poorly-selective (killing rapidly proliferating cells) and with high-spectrum (effective against most tumors). More new compounds have been added as possible tools for anti-cancer therapies, being characterized by a more selective mechanism and fewer side effects, resulting in better anti-cancer responses and/or fewer side effects. Nowadays, modern anti-cancer drugs are very selective (against a unique target) and, as a consequence, are effective only against tumor-bearing specific molecular abnormality (i.e., the higher selectivity, the lower antitumor spectrum). Recent advances in the knowledge of cancer biology led to the development of a new diagnostic and therapeutic arsenal that is not limited to selective target drugs but also includes already existing drugs that are multi-targeted or used in polypharmacology (Gonzalez-Fierro and Dueñas-González, 2021).

Drug repositioning is considered for several types of cancer. The collection of information regarding the anti-cancer effects of drugs commonly used for other diseases could be of help in identifying potential repositionable compounds. As far as thyroid cancer (TC) treatment is concerned, few studies took into account the potential repositioning of drugs.

TC is the most common endocrine cancer, which has raised concern due to its rapidly increasing prevalence. Annual incidence varies by sex, age, and geographical location (Palanca et al., 2022). The incidence of TC is the ninth highest in the world (Chen DW. et al., 2023a) and, during the last 40 years, increased globally. According to many studies, the increase in TC incidence is a consequence of the detection of small, low-risk papillary TC due to increased thyroid ultrasonography use. During the past 5–10 years, TC care experienced profound changes, with several therapeutic options now available (Palanca et al., 2022; Chen DW. et al., 2023a).

Most TCs are slow-growing and highly responsive to standard therapies which include thyroidectomy, and in selected cases radioactive iodine treatment (RAI), and TSH suppressive therapy (Haugen et al., 2016). Even if these approaches are successful in many cases, there is still a subset (≈3%–5% of patients) that progress to therapeutically refractive disease, constituting the so-called “TC-related deaths due to the lack of effective treatment.” In this view, some efforts are ongoing to identify personalized therapy for refractory patients and drug repositioning represents one of the potential alternatives (Xu et al., 2019). In particular, several studies suggested that some anti-diabetic drugs could be of potential interest because of their anti-cancer effects demonstrated both *in vitro* and *in vivo* on TC.

This review aims to overview the available studies regarding the multiple anti-cancer effects of anti-diabetic drugs discovered in TC as well as to discuss their potential repositioning for TC therapy. Moreover, an exhaustive description of drug repositioning including specific examples of anti-diabetic repositionable drugs for TC treatment will be also provided.

## Drug repositioning

Drug repositioning is an alternative approach to identify new fields of application for existing drugs, currently approved for a different clinical condition. The drug repositioning activity is based on *in vivo* and *in vitro* tests. Polypharmacology is the basis for drug repurposing: this principle asserts that a drug with multiple targets can have multiple mechanisms of action (Nowak-Sliwinska et al., 2019). Therefore, polypharmacology can be used in the search for more effective and less toxic treatments.

Drug repositioning could indeed present several advantages as compared to the development of a new drug. Once repositioned, information regarding the safety, pharmacology, and toxicology of a given drug already exists, being approved by the Food and Drug Administration (FDA) for clinical use in humans.

Drug repurposing is time- and cost-saving compared with “*de novo*” drug development. Indeed, the time from discovery to clinical trial averages 9 years, the success rate is less than 10% with elevated average cost. In contrast, drug repositioning can take 3–4 years to reach clinical trials (Sams-Dodd, 2005). However, in this case, there are numerous obstacles to cross, mainly financial and resource barriers, intellectual properties, data access barriers, biases, and liability risks (Masuda et al., 2020).

Numerous collaborative initiatives with the final aim of drug repurposing were objects of debate, among wich: The AZ Open Innovation program, the NIH National Center for Advancing Translational Sciences (NCATS) program: Discovering New Therapeutic Uses for Existing Molecules, the Medical Research Council (MRC) and AstraZeneca (AZ) Mechanisms of Human Disease Initiative, The Clinical and Translational Science Award (CTSA) Pharmaceutical Assets Portal, European College of Neuropsychopharmacology (ECNP) Medicines Chest Program, Pfizer’s SpringWorks Program, the AstraZeneca/National Research Program for Biopharmaceuticals (NRPB) partnership in Taiwan, the Roche/Broad Institute Collaboration, and the Drugs for Neglected Diseases Initiative (DNDI), the Clinical Development Partnerships Initiative (Krishnamurthy et al., 2022).

Some examples of drugs that have been repositioned for the treatment of certain types of cancer over the years include Sunitinib, a drug that was successfully repositioned for the treatment of gastrointestinal stromal tumors and renal cancers. In addition, in 2010, this compound was also approved for the treatment of pancreatic neuroendocrine cancers. Another drug Tamoxifen, that was originally known for its ability to increase fertility, was repurposed for breast cancer therapy. Moreover, it was proven to reduce breast cancer risk and approved also for this purpose. Tamoxifen was included in the standard of care for long-term adjuvant therapy for estrogen receptor-positive breast cancer (Krishnamurthy et al., 2022).

The main aim of drug repositioning is to counteract attrition and rising costs, which, according to “Eroom’s law,” have produced a large increase in the number of new drugs entering the pharmaceutical market. However, this approach must be considered as an add-on rather than an alternative to the search for novel drugs. (Jourdan et al., 2020).

In drug repositioning it is crucial to understand the disease-drug relationship, for this reason, there are several approaches to address the problem:

• In silico approaches: in this type of approach, various public databases, clinical trials, and bioinformatic systems are used to identify drug-target interactions (Ekins et al., 2007).
• Cluster approaches tend to find new drug-target or drug-disease relationships (Parisi et al., 2020).
• Propagation approaches: this approach allows the discovery of disease-gene, disease-disease, and disease-target relationships (Picart-Armada et al., 2019).
• Experimental approaches: including target screening, cellular assays (Shah et al., 2016), animal models (Ridges et al., 2012), and clinical studies (Zhu et al., 2014).

An important aspect of drug repositioning is to identify a compound that could also overcome the obstacle of drug resistance in cancer therapy. Therefore, it is necessary to follow strategies to minimize drug resistance and maximize antitumor activity (Wang et al., 2019).

## Anti-diabetic drugs repositionable as potential anti-cancer drugs

Diabetes Mellitus (DM) is a chronic disease that occurs either when the pancreas does not produce enough insulin or when the body cannot effectively use the insulin it produces. Insulin is a hormone that regulates blood glucose. In type 2 diabetes mellitus (T2DM), hyperglycemia is a common effect of uncontrolled diabetes and over time leads to serious damage to many of the body’s systems, especially the nerves and blood vessels. Hypoglycemic agents, also known as oral antidiabetics, represent a heterogeneous group of drugs that are used in the treatment of T2DM.

DM is characterized by elevated blood sugar (glycemic) levels, therefore the role of these drugs is to reduce glycemic levels, possibly avoiding hypoglycemic events. The other important aim of modern anti-diabetic therapy is to reduce mortality risk and long-term complications of T2DM, such as cardiovascular, renal and neurological damage. These drugs work by different mechanisms, some reduce blood glucose by increasing the secretion of insulin; others slow intestinal absorption of glucose, others increase renal elimination of glucose and others increase insulin sensitivity of target tissues. The classes of antidiabetics currently in use are summarized in Table 1.

**TABLE 1:** Commonly used anti-diabetic drugs.

Classes of antidiabetcs	Name	
Biguanides	-Metformin (in use)	
	-Phenformin (removed in 1970)	
	-Buformin (removed in 1970)	
Sulfonylureas	-Glibenglamide (in use)	
	-Glicazide (in use)	
	-Glimepiride (in use)	
Glinides	-Gepaglinide (in use)	
Thiazolidinediones	-Troglitazone (removed in 2000)	
	-Pioglitazone (in use)	
	-Rosiglitazone (removed in 2010)	
	-Ciglitazone (prototype, never used in clinical practice)	
	-Lobeglitazone (in use only in korea)	
DPP-4 enzyme inhibitors	-Sitagliptin (in use)	
	-Gempigliptin (in use)	
	-Linagliptin (in use)	
	-Saxagliptin (in use)	
	-Vildagliptin (in use)	
	-Alogliptin (in use)	
Inhibitors of the renal glucose transporter SGLT-2	-Canagliflozin (in use)	
	-Dapagliflozin (in use)	
	-Empagliflozin (in use)	
Glp-1 analogues	-Exenatide (in use)	
	-Liraglutide (in use)	
	-Semaglutide (in use)	
	-Dulaglutide (in use)	
	-Lixisenatide (in use)	
Insulin analogues	Fast acting	
	- Lispro (in use)	
	- Aspart (in use)	
	- Glulisine (in use)	
	Long acting	
	- Detemir (in use)	
	- Degludec (in use)	
	- Glargine (in use)	
	Intermediate acting	
	- Human insulin (in use)	
	- NPH (in use)	

The rationale behind the use of anti-diabetic drugs as potential candidates for drug repositioning in anti-cancer therapy, especially for TC, stems from several data. First of all, it is widely recognized that T2DM is a risk factor for developing several types of cancer, mainly colorectal, liver, pancreatic, and endometrial cancer (Ling et al., 2023). Some studies have also suggested a possible correlation between T2DM and TC (Aschebrook-Kilfoy et al., 2011; Yeo et al., 2014). Moreover, some authors have suggested that hyperinsulinemia, hyperglycemia, and chronic inflammation typical of untreated DM would be risk factors for TC development and/or progression (Jee et al., 2005; Giovannucci et al., 2010), and thus correcting them with anti-diabetic drugs would have indirect therapeutic effects for TC. Nevertheless, it must be acknowledged that some studies failed to report any association between T2DM and TC (Kitahara et al., 2012; Tse and ng, 2012; Seo et al., 2017). Moreover, the association observed in some epidemiological studies could be sustained by the fact that T2DM and TC could share some common risk factors, such as obesity and increasing age (Kitahara et al., 2020), and TC is often diagnosed incidentally in patients with T2DM due to the performance of screening imaging (such as carotid artery ultrasound), potentially leading to a selection bias (Croce et al., 2023).

The second reason why anti-diabetic drugs could be beneficial in TC is that TC cells often display abnormal metabolic pathways that could be sensitive to the specific action of anti-diabetic drugs. In particular, malignant cells can modify their energy metabolism in response to a challenging environment, allowing tumor cells to survive and spread (Coelho et al., 2018). Most malignant cells rely on glucose metabolism through aerobic glycolysis, the so-called Warburg effect, with a sharp increase in glucose uptake and lactate production (Lin et al., 2020). This metabolic shift has also relevant consequences beyond favoring cancer growth, such as influencing tumor microenvironment and vascular invasion. Some anti-diabetic drugs can interfere with intracellular metabolic regulation, for example, through activation of AMPK or inhibition of glucose uptake, favoring TC cell death and also reducing its aggressiveness.

### Biguanides

Biguanides are oral hypoglycemic drugs derived from the *Galega officinalis* plant, originally developed for the treatment of T2DM. The active components responsible for the effects were guanidines and galectins. In 1918, animal studies demonstrated that guanidine had hypoglycemic activity, but it was highly toxic for clinical use. Due to this reason, attention was focused on synthetic derivatives of these natural compounds. The first biguanide to be tested on humans in the XX century was metformin. In 1957, Jean Stern physician and clinical pharmacologist published the results of a study on the antidiabetic properties of several biguanides. Among them, dimethyl biguanide (known as metformin) was selected for clinical development with the suggestive name of “Glucophage,” which means “glucose eater.” The other biguanides, phenformin and buformin, were more potent than metformin, however in the late 1970s they were removed in most countries because of their association with lactic acidosis (Blough et al., 2015). Thus, among biguanides, metformin remains at present the most widely employed anti-diabetic drug. Of note, biguanides are not limited to be used as anti-diabetic drugs. Indeed, biguanide derivatives are used for multiple therapeutic purposes ranging from antimalarial (proguanil, cycloguanil), antiviral (monoxydine), anti-septic and disinfectant (chlorhexidine, alexidine, pycloxidine, polyhexanide) (Kathuria et al., 2021). Biguanides are extensively studied for their potential anti-cancer properties in several types of cancer including the thyroid one. The anti-cancer properties of biguanides are displayed through several mechanisms of action, including the activation AMPK pathway with the consequent inhibition of mTOR and reduction of several pro-tumorigenic effects including cell proliferation and production of inflammatory mediators (Seyfried et al., 2014; Luengo et al., 2017). Biguanides also interacts with REDD1, (Luengo et al., 2017), IRS receptors, (Luengo et al., 2017), glucose metabolism, (Luengo et al., 2017; IDF, 2021; WHO, 2023), oxidative phosphorylation, (Stine et al., 2022; Batta et al., 2020; Abudawood, 2019), lactic acid production, (Luengo et al., 2017), and cell cycle (Bell et al., 2023). Of note many other mechanisms are involved in the anti-cancer effects of biguanides, some of them more related to the specific cancer type, like, for example, the interaction of proguanil with EGFR in glioblastoma cells. Thus, due to the broad spectrum of action, biguanides are currently regarded as extremely interesting compounds.

### Metformin

Metformin is an effective hypoglycemic agent, recommended as a first-line oral therapy for T2DM. Metformin was first approved in Canada in 1972, and received subsequent FDA approval in the United States in 1995. Metformin exerts its anti-diabetic effects through several mechanisms, such as inhibition of hepatic gluconeogenesis, reduction of insulin resistance, and enhancement of peripheral glucose uptake. Among its many possible pharmacologic properties, also anti-cancer effects have been reported. (Kushchayeva et al., 2014). Indeed, both *in vitro* and *in vivo* studies showed that metformin exerts benefits in several types of cancer. Metformin was shown to display anti-cancer effects through its insulin-lowering activity, which may slow tumor proliferation in individuals with insulin resistance. Moreover, metformin targets the respiratory complex I of the electron transport chain in the mitochondria of preneoplastic and neoplastic cells, reducing energy consumption in the cell. Other mechanisms on which Metformin play an action are: mammalian target of rapamycin (mTOR, crucial to tumor cell metabolism), adenosine mono-phosphate-activated protein kinase (AMPK), mitochondrial glycerophosphate dehydrogenase (mGPDH), and the nuclear factor kB (NF-kB) (Chan, 2016). Importantly, some studies suggest that metformin could cooperate with chemotherapeutic drugs (Kushchayeva et al., 2014; Yoshida et al., 2020).

Controversially, emerging evidence suggest that metformin has potential clinical applications in stem cell medicine, regenerative medicine and anti-aging (Jiang and Liu, 2020). Indeed, several studies have demonstrated that metformin promotes osteogenic, neuronal, myogenic, and adipogenic differentiation with varying results (Zhan et al., 2020; Ould-Brahim et al., 2018; Dadwal et al., 2015; Ahn and Cho, 2017; Fatt et al., 2015; Smieszek et al., 2018). Metformin induces cell proliferation at low concentrations‚ but it has anti-tumor activity at higher concentrations and can inhibit the proliferation of cancer cells (Nashif et al., 2023). At present, research is limited to *in vitro* and experimental animal models, however it can be hypothesized that the contradictory effects of metformin might be dependent upon specific cell types and/or dose differences.

Results from clinical studies regarding the anti-cancer effects of metformin are rather controversial. Indeed, while some retrospective-case control studies, have shown that metformin is able to reduce overall cancer incidence and mortality by 10%–40% (IDF, 2021), RCTs failed to show a significant reduction in cancer incidence and mortality (Stevens et al., 2012; Wen et al., 2022). This discrepancy could be due, at least in part, to the short follow-up time and/or to the biological heterogeneity of cancer. Given its multiple pathways of action and its anti-cancer effects observed in different types of cancer, metformin is the anti-diabetic drug that was most studied in the field of TC. Here will follow a summary of the crucial studies that highlight the importance of metformin in the treatment of TC.

Given its multiple pathways of action and its anti-cancer effects observed in different types of cancer, metformin is the anti-diabetic drug that was most studied in the field of TC. Here will follow a summary of the crucial studies that highlight the importance of metformin in the treatment of TC.

Similarly, some authors observed that metformin users had a lower clinical severity of TC at presentation and a longer disease free survival in metastatic patients (Jang et al., 2015), while other authors did not observe any difference in the risk of developing metastases when compared with non-treated patients (Noh et al., 2019). Among the possible mechanisms contributing to the anti-tumoral effect of metformin in TC, a TSH-lowering effect can be observed in patients treated with metformin, for several clinical condition (Isidro et al., 2007; Rotondi et al., 2011; Cappelli et al., 2012; Cappelli et al., 2014). This effect would be specific for TC and coud play a role especially in combination with other therapies. Nevertheless, it must be acknowledged that available data all come from retrospective and non-randomized studies. High quality data coming from prospective randomized trials on the use of metformin, alone or in combination, in the treatment of TC are currently lacking.

Interesting findings emerged also from studies *in vitro* on different types of thyroid cells (human anaplastic, differentiated cancer cells, as well as human normal and cancer thyroid primary cell cultures, TC stem cells and rat follicular thyroid cells). These studies highlighted several anti-cancer effects exerted by metformin on TC cells including: i) induction of cell death, ii) reduction of cell growth, iii) inhibition of the metastatic potential iv) modulation of chemokines in the tumor microenvironment, v) de-differentiating effects (increase in iodine up-take) (Chen et al., 2012; Klubo-Gwiezdzinska et al., 2013; Cho et al., 2014; Moon and Mantzoros, 2014; Rotondi et al., 2015; Kim et al., 2018a; Klubo-Gwiezdzinska et al., 2012; Han et al., 2015; Kheder et al., 2017; Nozhat et al., 2018; Ye et al.; Sloot et al., 2019; Durai et al., 2021; Morale et al., 2022). The reduction of cell growth and viability showed in TC cells were due to the regulation by metformin of the AMPK-mTOR pathway, inhibiting cell cycle progression and inducing apoptosis (Klubo-Gwiezdzinska et al., 2012; Klubo-Gwiezdzinska et al., 2013; Thakur et al., 2019). Other mechanisms explaining the effects of metformin on thyroid cell growth and viability are the downregulation of cyclin D1, the increase of the ER stress and the reduction of mRNA levels of AKT, PI3K, and FOXO1 LRP2 and p-JNK, genes that play a crucial role in cell proliferation and survival (Oyadomari and Mori, 2004; Bikas et al., 2015; Nozhat et al., 2018; He et al., 2020). Moving to the ability of metformin to reduce the metastatic potential, it was demonstrated that the drug not only reduced the migration of MTC cancer cells (Klubo-Gwiezdzinska et al., 2012), but also modified the expression of several markers of the epithelial-tomesenchimal-transition (EMT) (Han et al., 2015). A further interesting anti cancer effect of metformin was the ability to reduce the TNF-α-induced CXCL8 secretion by thyroid cells *in vitro*. CXCL8 is a protumorgenic chemokine, thus, this CXCL8-lowering effect of metformin was considered as a further indirect anticancer property of the drug. Metformin was also shown to play a potential role in the re-differentiation of TC cells. Indeed, undifferentiated TC cells express low levels of sodium/iodine-symporter (NIS), a protein membrane crucial for iodine uptake by thyrocites and consequently essential for the efficacy of RAI, the most effective therapy for TC in case of not complete surgical removal. A recent study in ATC cells showed that metformin increased NIS mRNA and protein expression (as well as mRNA of thyroglobulin, TSHR, and NKX2.1) acting also as a demethylating agent (Durai et al., 2021).

Finally, some studies also showed that metformin could synergize with other drugs increasing their anti-cancer effects. Indeed metformin showed synergic effects with Sorafenib (a multikinase inhibitor) (Chen et al., 2015), with vemurafenib (a selective inhibitor of BRAFV600E), with gemigliptin (dipeptidyl peptidase-IV inhibitor) (Kim et al., 2018a) and pioglitazone (a TDZ) (Kim et al., 2018a; Ozdemir Kutbay et al., 2020) in several TC models (Hanly et al., 2015; Durai et al., 2021).

These *in vitro* studies make metformin desirable for potential repositioning as anti-cancer compounds in the treatment of TC, thus several studies are still ongoing to deeply characterize its effect.

### Phenformin

In the late 1950s, Phenformin was introduced in the United States for the treatment of non-insulin-dependent (NIDDM) and was 50 times more powerful than metformin. Despite this, it was removed from the market in the late 1970s because of its high risk of lactic acidosis (Luft et al., 1978). The higher efficacy of phenformin compared with metformin seems to be due to different modes of entry into cells. Indeed, the fact that administration of phenformin can induce lactic acidosis, while metformin does not, suggests that these two biguanides act through different pathways (García Rubiño et al., 2019).

Various *in vitro* and *in vivo* studies performed in different types of tumors, highlighted the ability of phenformin to reduce cancer cell proliferation strongly than metformin (Janzer et al., 2014).

Up to now, only one study demonstrated the ability of phenformin to reduce cell viability in TC cells. Moreover, phenformin, at non-cytotoxic concentrations, had also an indirect anti-cancer effect through modulation of the chemokine milieu within the thyroid tumor microenvironment (inhibition of CXCL8 secretion) (Rotondi et al., 2018). These data expand the potential benefits of this molecule as an antitumor drug *in vivo* (Rotondi et al., 2015). Furthermore, phenformin could also display synergism with other anti-cancer molecules (i.e., immune-therapeutics, chemotherapeutic).

In 2011, a study revealed that the administration of phenformin in combination with 2-deoxyglucose can prevent the development of lactic acidosis, reducing toxicity. Therefore, in terms of cancer therapy, perhaps phenformin should be reevaluated (Lea et al., 2011).

As far as thyroid cancer is concerned, *in vitro* and *in vivo* studies focused on the potential anti-cancer effects of metformin and phenformin. A parallel of the effect reported for two biguanides (metformin and phenformin) in thyroid cancer and other solid cancer should be overviewed. Besides the well-known targets such as the inhibition of the mTOR signaling pathway, several additional metformin and phenformin targets (e.g., mGPDH, ATF3, STAT3, GSK3, cyclins) have been identified in other cancers (Thakur et al., 2019). Induction of cell cycle arrest, reduction of viability and induction of ROS were observed in several types of cancers by both drugs. Thus, the anti-cancer effects of both compounds appear to be exerted through several modes of action (action on AMPK, cell cycle, mytocondrial complex-I), likely occurring without a cancer type specificity.

On the other hand, other biguanides (not limiting to anti-diabetic compounds) were tested for their potential anti-cancer properties in different types of cancer. Just to give few examples, buformin and phenformin were showed to inhibit the viability of pituitary cancer cells *in vitro* (Vázquez-Borrego et al., 2019). Chemical modification of metformin into sulfenamides and sulfonamides has also improved the cellular accumulation of these compounds in cancer cells, with a subsequent increase in their cytotoxic efficacy. Many sulfonamide derivatives of metformin exerted cytotoxicity in human breast cancer cells (MCF-7 or MDA-MB-231, or both) induced particularly by methylated phenyl sulfonamides and was associated with their ability to arrest the cell cycle in the G0/G1 phase and subsequently to cause apoptosis (Torunoglu et al., 2023). The novel biguanide MC001 showed much stronger antitumor effect and relatively weaker proglycolytic activity compared with metformin as shown *in vitro* in colorectal cancer cells (Fu et al., 2022). In breast cancer cell lines, it was shown that Cycloguanil and its most promising analogue, NSC127159, were shown to inhibit Dihydrofolate reductase (DHFR), an established anti-cancer drug target whose inhibition disrupts folate metabolism and STAT3-dependent gene expression. A very recent study demonstrated that novel biguanide derivative, IM176, induces prostate cancer cell death *in vitro* by modulating the AMPK-mTOR and androgen receptor signaling pathways (Kim et al., 2023). A study on glioblastoma stem cells reported that phenformin, moroxydine, proguanil and cycloguanil exerted a significant impairment of glioblastoma stem cells proliferation, invasiveness and self-renewal (Barbieri et al., 2018). In 2015, Wysham et al., performed a comparative study of metformin and the novel biguanide NT1014 demonstrating anti-proliferative activity of metformin and NT1014 in ovarian cancer cell lines by inducing cell cycle arrest in G1 phase followed by apoptosis. *In vivo*, NT1014 reduced tumor weight by 61%, whereas metformin by only 32%. A study on bladder cancer showed that proguanil induces autophagic death of BC cells by specific binding to EGFR and inhibiting its expression.

Taken together the anti-cancer effect of biguanides is being studied in several types of cancer suggesting the potential repurpositioning not only of the anti-diabetics, but also of numerous other biguanides-related compounds.

### Sodium-glucose transporters (SGLT)-inhibitors

The treatment of T2DM with glyflozines, sodium-glucose co-transporter 2 inhibitors, represents a new therapeutic approach (Devineni and Polidori, 2015). These classes of inhibitors act by decreasing renal glucose uptake and increasing urinary glucose excretion. SGLT2 is located in the initial segment of the proximal tubule, and is responsible for 80%–90% of reabsorption, while SGLT1s reabsorb the remaining 10%–20% (DeFronzo et al., 2017).

Among SGLT2 inhibitors, three of them have been approved by the FDA and EMA: canagliflozin (2013), dapagliflozin (2014) and empagliflozin (2014); three other compounds have been approved in Japan (Ipragliflozin, Tofogliflozin, Luseogliflozin), while others are in development (Ertugliflozin and Sotagliflozin) (Devineni and Polidori, 2015). Recent studies have highlighted that SGLT2 inhibitors, including canagliflozin and Dapagliflozin, can inhibit cancer and colorectal cell growth through inhibition of SGLT2-mediated glucose uptake (Dutka et al., 2022).

### Canagliflozin

Canagliflozin [(1 *S*)-1,5-andro-1-[3-[[5-(4-fluorophenyl)-2-tienil]metil]-4-metilfenil]-d- GLUCITOLO emirate], is a C-glycosyl compound that is used (in the hemihydrate form) for the treatment of T2DM through inhibition of sodium/glucose co-transporter 2 (Choi, 2016) and was approved by the FDA in 2013. Canagliflozin is an orally active selective SGLT2 inhibitor. It is administrated orally and is rapidly absorbed, reaching peak plasma concentration in 1–2 h (Devineni and Polidori, 2015). Canagliflozin acts both delaying intestinal glucose absorption as well as increasing urinary glucose excretion; this mechanism contributes to lower postprandial blood glucose.

It was initially approved by the FDA in 2013 for the management of T2DM and later approved in 2018 for a second indication of reducing the risk of cardiovascular events in patients diagnosed with T2DM. Recently, it was observed that canagliflozin promotes AMPK activity by inhibiting mitochondrial respiration in embryonic kidney cells and mouse cells (Hawley et al., 2016). As with biguanides, canagliflozin inhibits mitochondrial respiration and cell proliferation, suggesting that it may be useful in cancer prevention and treatment.

Studies *in vitro* and *in vivo* have demonstrated the inhibitor effect of canagliflozin on TC cells. The SGLT2 inhibitor could suppress the glycolysis level of TC cells and also interfere with glucose uptake and glycolysis in TC cells. In addition, the treatment with canagliflozin induced cell apoptosis of TC cells (Wang et al., 2022). Furthermore, this anti-diabetic drug increases the activation of ATM/CHK2 in TC cells, indicating DNA damage repair is initiated. In addition, recent studies have shown that Canagliflozin is also able to activate AMPK, through inhibition of complex I of the respiratory chain. This suggests that some therapeutic benefits of canagliflozin could result from the activation of AMPK, rather than inhibition of SGLT2 (Hawley et al., 2016). Thus, further studies are needed to evaluate Canagliflozin as a candidate for repositioning as an anticancer agent, including TC.

### DPP-4 inhibitors

These compounds, by preventing deactivation by dipeptidyl peptidase-4 (DPP4) inhibitors, improve the concentration of endogenous incretins (Ahrén et al., 2002; Vangoitsenhoven et al., 2012). Currently, DPP-IV inhibitors are widely used as monotherapy or combination therapy for the treatment of patients with T2DM.

DPP-IV modulates diverse cellular processes including survival, proliferation, and differentiation, and thereby enhances or diminishes tumorigenesis depending on the types or phases of tumors (Havre et al., 2008). In this regard, DPP-IV can be either overexpressed or underexpressed in human solid tumor tissues, suggesting the possible role of DPP-IV as a potential diagnostic marker and therapeutic target in solid tumors (Havre et al., 2008).

Initially, a register-based study from Taiwan suggested that the administration of DPP-4 inhibitors could be associated with an increased risk of TC (Tseng, 2016). On the other hand, the investigation of DPP-4 inhibitors as potential anti-cancer agents was not discouraged by this study because DPP4 expression in TC was demonstrated to be associated with cellular invasion, promoting TC cell metastasis, and a more aggressive disease in papillary TC (Lee et al., 2017; He et al., 2022). More interestingly a recent *in vitro* study showed that DPP4 gene silencing inhibits papillary TC cell proliferation and EMT and promotes cell apoptosis (Hu et al., 2021). In addition, in contrast with previous findings, a systematic review and metanalysis by Overbeek et al. (2018), showed that it is not possible to conclude whether DPP-4 inhibitors were associated with an increased risk of site-specific cancer including TC. Another population-based cohort study of patients with T2DM with a concomitant cancer showed that no increased risk of metastasis was associated with DPP-4 inhibitor therapy (Noh et al., 2019). Thus, the targeting of DPP4 was considered as a potential therapeutic strategy for DPP4-expressing TC and further studies were encouraged.

#### Sitagliptin

Sitagliptin is an oral dipeptidyl peptidase-4 (DPP-4) inhibitor used in conjunction with diet and exercise to improve glycemic control in patients with T2DM. The effect of this medication leads to glucose-dependent increases in insulin and decreases in glucagon to improve control of blood sugar. Sitagliptin was granted FDA approval on 16 October 2006 (National Center for Biotechnology Information, 2023f).

Tseng (2016) showed that among Taiwanese patients with T2DM, sitagliptin use may be associated with an increased risk of TC. On the other hand, this compound showed a reduction of TC cell viability, proliferation, and some aspects related to the metastatic process *in vitro*. Indeed, Hu et al. (2021) reported cytotoxic effects of Sitagliptin on TC cell lines TPC-1 and GLAG-66 as well as a reduction of cell proliferation in both TC cell lines. Interestingly, Sitagliptin was able to reduce TC cell migration by influencing the expression of some markers of the epithelial-to-mesenchimal-transition (EMT) which ultimately drives cancer cell migration (Hu et al., 2021). In addition, a recent *in silico* molecular docking study regarding the *DPP4/CTNNB1/MET* signatures showed that stagliptin could be a potential TC drug, however more investigations are surely needed to confirm it (Cheng et al., 2022).

#### Gemigliptin

Gemigliptin is an orally bioavailable inhibitor of DPP-4, with hypoglycemic and potential renoprotective activities. Upon administration, gemigliptin binds to DPP-4 and inhibits the breakdown of the incretin hormones, glucagon-like peptide-1 (GLP-1), and glucose-dependent insulinotropic polypeptide (GIP). This prolongs incretin activity, increases postprandial insulin secretion from pancreatic beta cells, decreases glucagon secretion, delays gastric emptying, and lowers blood glucose levels (National Center for Biotechnology Information, 2023a).

In 2017, Kim et al. (2017) demonstrated that gemigliptin was able to induce TC cell death *in vitro*. Another study of the same group further demonstrated the cytotoxic *in vitro* effect of gemigliptin in TC cells also showing an increase in its cytotoxic activity when combined with one histone deacetylase inhibitor (PXD101) (Kim et al., 2018b). More interestingly, these investigators showed that gemtigliptin in combination with another anti-diabetic compound, but belonging to a different class (biguanides), Metformin (Kim et al., 2018a), exerts a stronger adverse effect on TC cells *in vitro.* Indeed, TC cells treated with both gemigliptin and metformin showed synergistic cytotoxicity of two agents, exerted by acting on Akt and AMPK pathways. The same study also showed that gemigliptin increased the inhibition of cell proliferation and migration induced by metformin by involving of ERK,MMP-2-9, p53, p21, VCAM-1, and (Kim et al., 2018a).

### Thiazolidinediones

Thiazolidinediones (TZDs), (also called “glitazones”) were introduced in 1996 for T2DM, when troglitazone (Rezulin; Parke-Davis/Warner-Lambert) was approved by the Food and Drug Administration. TZDs uniquely target insulin resistance, which is a core physiologic defect in T2D, and significantly improve glucose control. Unfortunately, due to severe hepatic and cardiovascular side-effects, most TZDs were removed from the clinical use, with on pioglitazone still recommended by most guidelis as an anti-diabetic drug. However, TZDs improve insulin action in adipose, hepatic tissue and muscle, agonizing with of peroxisome proliferator–activated receptor-γ (PPAR-γ) nuclear receptors. PPAR-γ activation is translated into different vascular and metabolic effects including the upregulation and downregulation of different genes essential for e lipid and glucose metabolism, but also for inflammatory response. *In vitro* data highligted that PPAR*γ* could be suggested as targets for TC therapy (Chung et al., 2002; Martelli et al., 2002; Hayashi et al., 2004).

#### Troglitazone

Troglitazone was the first TZD approved for use in the United States and was licensed for use in T2DM in 1997, but withdrawn 3 years later because of the frequency of liver injury, including acute liver failure, associated with its use. Troglitazone has several recognized therapeutic properties as a hypoglycemic agent, an antioxidant, a vasodilator agent, an anticonvulsant, an anticoagulant, a platelet aggregation inhibitor, an antineoplastic agent, an EC 6.2.1.3 (long-chain-fatty-acid--CoA ligase) inhibitor and a ferroptosis inhibitor (National Center for Biotechnology Information, 2023h).

A first *in vitro* evaluation of the potential anti-cancer effects of troglitazone showed that the compound was efficient in inducing re-differentiation of TC cells *in vitro*, enhancing RAI-uptake. Subsequent studies showed that troglitazone inhibited anaplastic TC cell proliferation *in vitro* and increased the effect of paclitaxel (Copland et al.).

Combined treatment with ovastatin and lovastatin (a cholesterol-lowering agent) inhibited epidermal growth factor-induced migration of anaplastic TC cells (Chin et al., 2017). Moreover, an *in vitro* and *in vivo* mouse model of anaplastic TC showed that troglitazone + Lovastatin display anti-cancer effects such as reduction of cell proliferation and tumor regression (Zhong et al., 2018).

#### Pioglitazone

Pioglitazone is both a PPARα and PPARγ agonist with hypoglycemic activity and an insulin-sensitizing role.Pioglitazone is the only drug of the TZD class still commonly used in clinical practice. Moreover, it is recognized to be a pantothenate kinase inhibitor, a long-chain-fatty-acid--CoA ligase inhibitor, a ferroptosis inhibitor, a cardioprotective agent, an antidepressant, and a geroprotector (National Center for Biotechnology Information, 2023g).

Several studies reported that pioglitazone could exert benefits against TC.

Indeed, it was shown that Pioglitazone increased the iodide uptake *in vitro* by thyroid cells (Fröhlich et al., 2005) and exerted a reduction of cell proliferation in anaplastic TC cells *in vitro* (Antonelli et al., 2009). Pioglitazone was demonstrated to induce cellular lipid accumulation in TC cells *in vitro*. Moreover, it was demonstrated that TF-1 interacts with PPFP to inhibit the pro-adipogenic response to pioglitazone and that the ability of pioglitazone to decrease TTF-1 expression contributes to its pro-adipogenic action (Xu et al., 2016). Ozdemir Kutbay et al. (2020) showed that the combination of metformin and pioglitazone induced significant reductions in the level of oncogenic genes (AKT3, DEPTOR, EIF4E, ILK, MTOR, PIK3C, and PRKCA) in TC cells. This finding would indicate that TC progression could be prevented and these genes could be selected as therapeutic targets (Ozdemir Kutbay et al., 2020).

Interesting data regarding the potential anti-cancer activity of pioglitazone come from studies performed with a transgenic mouse model characterized by a PAX8-PPARγ fusion protein (PPFP) (found in 30% of follicular thyroid carcinomas). This particular fusion confers oncogenic capacity in transgenic mice. A 2011 *in vivo* study demonstrated that, in this mouse model, the administration of Pioglitazone induces a proadipogenic antitumor response, with the final result of preventing metastasis and reducing tumor size (Dobson et al., 2011). Another 2017 study in the same mouse model showed that pioglitazone exerted the induction of infiltration of immune cells (macrophages and T cells) only in the presence of PPFP (Zhang et al., 2017) highlighting the importance of the use of this compound in that specific clinical setting. Indeed, a subsequent clinical trial showed that pioglitazone may be therapeutic in patients with TC bearing PPFP (Giordano et al., 2018). Among the available clinical data in human subjects, in 2012 one case report showed that pioglitazone treatment could have some positive effects in radioiodine-negative and progressive DTC patients (Rosenbaum-Krumme et al., 2012). Moreover, Tseng (2014a) showed a null association between pioglitazone use and TC risk in patients with T2DM.

Finally, a comprehensive study on diagnosis, prognosis, and potential drug screening for papillary thyroid carcinoma (PTC), based on five hub lncRNAs, identified pioglitazone among the potential drugs that could be effective for TC treatment (Li et al., 2021).

#### Rosiglitazone

Rosiglitazone was marketed both alone (Avandia) (National Center for Biotechnology Information, 2023b) and combined with metformin (National Center for Biotechnology Information, 2023e) (Avandamet) or with glimepiride (National Center for Biotechnology Information, 2023d) (Avandaryl). Like other TZDs, activates PPARs and is a selective ligand of PPARγ with no PPARα-binding action. Rosiglitazone display well known effect on insulin resistance, but also shows anti-inflammatory effects (Lombardi et al., 2008).

Several studies suggest that rosiglitazone could have an anti-cancer effect on TC. Rosiglitazone inhibited anaplastic TC cell proliferation *in vitro* and increased the effect of the chemotherapy paclitaxel (Copland et al.). In the study by Aiello et al. (2006) performed *in vitro* on anaplastic thyroid cells, it was demonstrated that the treatment with rosiglitazone reduced anchorage-dependent and -independent growth and migration of TC cells, and increased apoptosis rate by reducing Bcl-X(L) expression and caspase-3 and -7 activation. The effect of rosiglitazone on cellular growth was associated with cell cycle arrest and with an increase of cyclin-dependent kinase inhibitors p21 (cip1) and cyclin-dependent kinase regulator p27 (kip1), a decrease of cyclin D1, and inactivation of Rb protein. Finally, rosiglitazone increased the expression of thyroid-specific differentiation markers (Aiello et al., 2006). In an *in vitro* study, under normoxic or hypoxic conditions, it was reported that rosiglitazone inhibited TC cell growth and increased NIS protein expression. This data is of further support the ability of rosiglitazone to induce re-differentiatio of TC cell (Chen et al., 2020). Finally, a recent study showed that rosiglitazone significantly inhibited transforming growth factor-beta1 (TGF-β1)-induced EMT-associated processes such as fibroblast-like morphological changes, EMT-related protein expression, and increased cell migration and invasion in BCPAP and K1 TC cells. Furthermore, rosiglitazone suppressed TGF-β1-induced MMP-2 expression and phosphorylation of p38 MAPK, but not ERK1/2 (Jin et al., 2021).

The possible role of rosiglitazone as anti-cancer agent in TC was also investigated in the clinical setting. Philips et al. showed an increase in RAI-uptake upon treatment with rosiglitazone (Philips et al., 2004). The successful induction of RAI uptake (decreased thyroglobulin levels and decreased in tumor size) after treatment with rosiglitazone was showed in two studies performed in metastatic DTC patients (Elias and Lizotte, 2006; Elola et al., 2011). In addition an increased RAI-uptake in therapeutic ^131^I scans (Kebebew et al., 2006; Tepmongkol et al., 2008) was reported in a phase II clinical trial that on the other hand concluded a not complete response of patients (Eisenhauer et al., 2009; Kebebew et al., 2009). It should be acknowledged that these studies had several limitations, including the limited accuracy of the technique of 131I scans and the unknown status of receptor expression of the treated TC. The status of a currently ongoing trial (NCT 00098852) with rosiglitazone is not known.

In 2013 Tseng et al. by using the National Health Insurance (NHI) reimbursement databases of Taiwan showed that rosiglitazone use may reduce the risk of TC in patients with T2DM (Tseng, 2013).

#### Ciglitazone

Ciglitazone born in 1980 and is considered to be the prototypical of TZDs, indeed was never used as a medication. Several analogs were later developed, including pioglitazone and troglitazone. Ciglitazone also exerts anti-inflammatory activity through the modulation of nuclear factor-kappaB-mediated pathways. In addition, this agent inhibits angiogenesis by reducing vascular endothelial growth factor (VEGF) production and inhibits the growth of melanoma cells by inhibiting the expression of (C-X-C motif) ligand 1 (CXCL1) (National Center for Biotechnology Information, 2023c).

In the *in vitro* study by Martelli et al. (2002), it was demonstrated that the treatment with ciglitazone inhibited the growth of several types of thyroid carcinoma cell lines *in vitro* in a time-dependent manner. Moving to anaplastic TC, an *in vitro* study demonstrated that in a panel of six anaplastic thyroid cancer (ATC) cell lines, the treatment with ciglitazone reduced anchorage-dependent and -independent growth and migration, and increased the apoptosis rate of TC cells (Aiello et al., 2006). Another *in vitro* study showed that ciglitazone induced apoptosis of TC cells by affecting the cytochrome-c caspase 3 and PTEN-Akt pathways, in addition the necrosis was obtained by affecting the PARP pathway (Chen et al., 2006).

#### Lobeglitazone

Lobeglitazone activates PPAR-γ and promotes the binding of insulin at fat cells, reduces blood sugar levels, lowers hemoglobin A1C (HbA1C) levels, and improves lipid and liver profiles (National Center for Biotechnology Information, 2023i).

Only one study showed that TC cell lines treated with lobeglitazone *in vitro* showed a significant reduction of TGF-β1-induced EMT-associated processes and EMT markers expression reducing also cell migration and invasion. Moreover, the treatment with lobeglitazone restored TGF-β1-induced loss of E-cadherin, as observed using immunocytochemistry, and suppressed TGF-β1-induced MMP-2 expression and phosphorylation of p38 MAPK, but not ERK1/2 (Jin et al., 2021).

## Anti-diabetic drugs repositioning for tumor treatment: the other face of the coin

The previous chapter is suggestive of a collective practicable repositioning of most of the anti-diabetic drugs, in particular for the treatment of TC. On the other hand, not all anti-diabetic drugs are free from potential side effects, which makes them not suitable for repositioning therapy of TC. In particular, a specific class, GLP-1 analogs deserves to be discussed given their potential pro-tumorigenic effects in TC.

### GLP-1 analogues

Glucagon-like peptide-1 (GLP-1) receptor agonists are effective treatments for T2DM which lower glucose concentrations without weight gain (often with weight loss) and with low risk for hypoglycemia (Hinnen, 2017).

GLP-1-based therapies represent a significant advance in the treatment of T2D. One of these medications is liraglutide. FDA in 2014 approved the higher dose version of this compound (known as Saxenda) for chronic weight-management treatment. Oher GLP-1 like lixsenatide (Sanofi Aventis, trade name Lyxumia) and albiglutide (GlaxoSmithKline, trade names Epezan and Tanseum) are currently approved or are under consideration for diabetes treatment (Ladenheim, 2015). About the prescription of exenatide and liraglutide, both compounds are contraindicated in MTC patients or multiple endocrine neoplasia syndrome 2 (MEN 2) patients due to the increased incidence of C-cell hyperplasia and tumors combined with elevated calcitonin levels in preclinical studies in rodents. However, these observations have not been replicated in nonhuman primates or humans and are believed to be a rodent phenomenon due to the higher density of GLP-1R on rodent C cells, so the responses obtained on rodents may not be relevant to primates (Bjerre Knudsen et al., 2010; Samson and Garber, 2013; Drab, 2016).

On the other hand, no case report describing medullary thyroid carcinoma has been published in a patient being treated with a GLP-1 receptor agonist who had a morphologically normal thyroid and low calcitonin concentrations before such treatment. Efficient surveillance of an extremely large number of patients would be required to confirm or reject such a report (Nauck, 2013). Regarding this class of molecules, studies in rats have led to mixed results. Some studies conducted in rodents, with exanatide and liraglutide, showed an association regarding the development of thyroid C-cell tumors after long exposures to overtherapeutic doses (Bjerre Knudsen et al., 2010). Liraglutide-induced C-cell hyperplasia and C-cell adenomas in mice and rats, and was also associated with a significant increase in C-cell carcinomas in rats and female mice administered the highest liraglutide dose tested (Aroda and Ratner, 2011). Studies with exenatide showed an increase in the incidence of C-cell adenomas in rats (female), exposed to 130-fold higher than the clinical dose of exenatide. Of note, no effect on C-cell was observed in mice similarly treated (Bethel et al., 2019). In contrast to results obtained in rodents, *in vivo* studies in cynomolgus monkeys administering liraglutide showed no effect on the relative fraction of C cells in the thyroid gland after 87 weeks. The risk of TC associated with liraglutide has been examined in rodent and non-human primate animal model studies. Indeed the liraglutide long-term treatment has been associated with thyroid C-cell hyperplasia and tumors in rodents, but not in monkeys.

Semaglutide has received an official box warning for thyroid C-cell tumors in the United States. This caution is based only on data from rodent studies and is not unique for semaglutide amongst the GLP-1RA (Bjerre Knudsen et al., 2010; Pyke and Knudsen, 2013). In contrast to liraglutide (Bjerre Knudsen et al., 2010) and lixisenatide (European Medicines Agency, 2012) in rats, the drug dulaglutide did not show an increase in thyroid C-cell tumors in rats. However dulaglutide doses greater than 0.5 mg/kg were demonstrated to increase hyperplasia of thyroid C cells (Byrd et al., 2015).

These data suggest that rodents are particularly sensitive to the effects of GLP-1 agonists on thyroid C cells but these foindings could not be considered as predictive of an increased risk of thyroid C-cell tumors in patients under GLP-1 receptor agonist therapy (Byrd et al., 2015).

## Insulin

Insulin is a widely prescribed glucose-lowering agent (Sims et al., 2021) especially in patients affected by type 1 diabetes mellitus (T1DM) as well as in some patients with Type 2 diabetes mellitus. The potential cancerogenic property of insulin is among the safety concerns related with long-term insulin therapy. Indeed, insulin is a growth factor, and the administration of exogenous insulin could, at least theoretically, stimulate tumour growth (Karlstad et al., 2013). The oncogenic effect of insulin could be due to the overexpression of insulin receptor by cancer cells, but also to its ability to interact with the IGF1 receptor, especially at supraphysiologic doses (Baricevic et al., 2015; Gallo et al., 2018) and with the use of long-acting analogues (Sciacca et al., 2010). Indeed, several studies have demonstrated that aberrant IGF signaling plays a critical role in the pathogenesis and progression of several types of cancer, including lung, breast, colon, prostate, ovary, pancreas, and thyroid (Bowers et al., 2015).

Nevertheless, data coming from observational studies are still conflicting and inconclusive, since some authors observe an association between insulin therapy and increased cancer risk (Currie et al., 2009; Tseng, 2019; Vicentini et al., 2022), while others failed to register any association (Pocock and Smeeth, 2009; But et al., 2017; Tan et al., 2017).

The possible effect of insulin therapy as a risk factor for thyroid cancer comes from pre-clinical data suggesting that insulin signaling is a key mediator in thyroid cancer cell growth. Indeed, early *in vitro* studies on rat thyroid follicular cells showed that concurrent treatment with insulin and TSH significantly increased the cell number compared to treatment with TSH alone (Tramontano et al., 1986).

Moreover the IGF1 axis, which can be stimulated by excessive levels of circulating insulin, is one of the key pathways involved in proliferative responses in both normal and neoplastic thyroid cells (Vella et al., 2001; Malaguarnera et al., 2012; Vella and Malaguarnera, 2018; Manzella et al., 2019). Thyroid cancer cells also over-express the insulin receptor (IR), especially isoform IR-A. IR/IGF-1 receptor hybrids and IR-A lead to an over-activation of the IGF pathway, causing an enhanced mitogenic signaling and cancer development (Malaguarnera et al., 2011).

Only few clinical studies up to now evaluated the relationship between insulin therapy and thyroid cancer risk (Kushchayeva et al., 2022). A 2014 studies based on data from the reimbursement databases of all Taiwanese diabetic patients from 1996 to 2009 evaluated the incidence of thyroid cancer according to the use, duration and dosage of therapy with human insulin. The results failed to show any significant association between human insulin use and risk of developing thyroid cancer, even at higher doses (Tseng, 2014b). Similarly, the study by Luo et al. (2016), did not show any association between insulin use and incidence of thyroid cancer.

Most clinical data on insulin signaling in thyroid cancer derive from the hypothesis that insulin-resistance, typical of obesity, metabolic syndrome and T2DM, could be a risk factor for thyroid cancer development (Malaguarnera et al., 2017). The only evidence of a possible positive correlation between insulin use and thyroid cancer comes from a 2011 study analyzing data from the Danish National Diabetes Register and Cancer Registry. The aim of the study was to evaluate if diabetes status, duration of diabetes and insulin use could be risk factors for the development of several types of cancer. The results showed that cancer incidence was higher among diabetic patients using insulin versus non-users. When specifically evaluating thyroid cancer, a significant difference between insulin users versus non-users was observed only in female patients. The risk also increased in relation to disease and therapy duration (Carstensen et al., 2012).

In conclusion, although pre-clinical data would support a role of insulin therapy as a risk factor for thyroid cancer, clinical data are still inconclusive.

## Insulin secretagogues

The primary action of secretagogues is to increase the release of insulin by inhibiting ATP-sensitive potassium channels in the pancreatic β-cell membrane. These compounds are classified as sulfonylureas or non-sulfonylureas (glinides) (Davies, 2002). The sulfonylureas have been extensively used to treat type 2 diabetes for nearly 50 years, representing the second and most used oral hypoglycemic drugs after metformin. A first-generation including Tolbutamide, Acetohexamide, Carbutamide, Chlopropamide, and Tolazamide, was introduced in Germany since the 1950s. In the 1980s more potent second-generation sulfonylureas became available (glibenclamide, glibornuride, gliclazide, glipizide, and gliquidone). Lastly, glimepiride, a third-generation sulfonylurea, was introduced in 1995 in the United States (Tan and Nelson, 1996).

Glinides are insulin secretagogues that lack the sulfamide group of the sulfonylureas and differ from sulfonylureas in receptor affinity, binding sites, duration of action and mechanism of absorption and elimination (Culy and Jarvis, 2001). Three glinides have been approved for use: repaglinide, nateglinide, and mitiglinide.

According to data from several meta-analyses, an overall increased cancer risk was reported in patient using sulfonylureas compared with those treated with metformin or other diabetes medications (Wu et al., 2015; Sacks et al., 2018; Mekuria et al., 2019; Chen Y. et al., 2023b). A meta-analysis of 8 studies (3 cohort studies, 3 case-control studies and 2 clinical trials) failed to demonstrate any association between glinides and risk of cancer (Wu et al., 2015).

As concern TC, a recent study by Tseng et al. suggest that among the anti-diabetic agents, only sulfonylurea, and not insulin, was significantly associated with higher risk of TC (Tse and ng, 2012). Hyperinsulinemia or insulin resistance alone might not be responsible for thyroid cell proliferation in patients with type 2 diabetes. A possible explanation could be related to the different effects of insulin on the thyroid gland. Insulin may increase thyroid hormone transcriptional action and reduce TSH level probably through the effect of hypoglycemia on pituitary-thyroid secretory activity (Hu et al., 1994; Schultes et al., 2002). On the other hand, first-generation sulfonylureas have been well known to exert anti-thyroidal effects and may be goitrogenic in animals or human (Hershman and Konerding, 1968; NIKKILA et al., 1960). It is possible that higher level of TSH, even within the normal range, may increase the risk of TC (Kim et al., 2013).

## Conclusion

**FIGURE 1:**
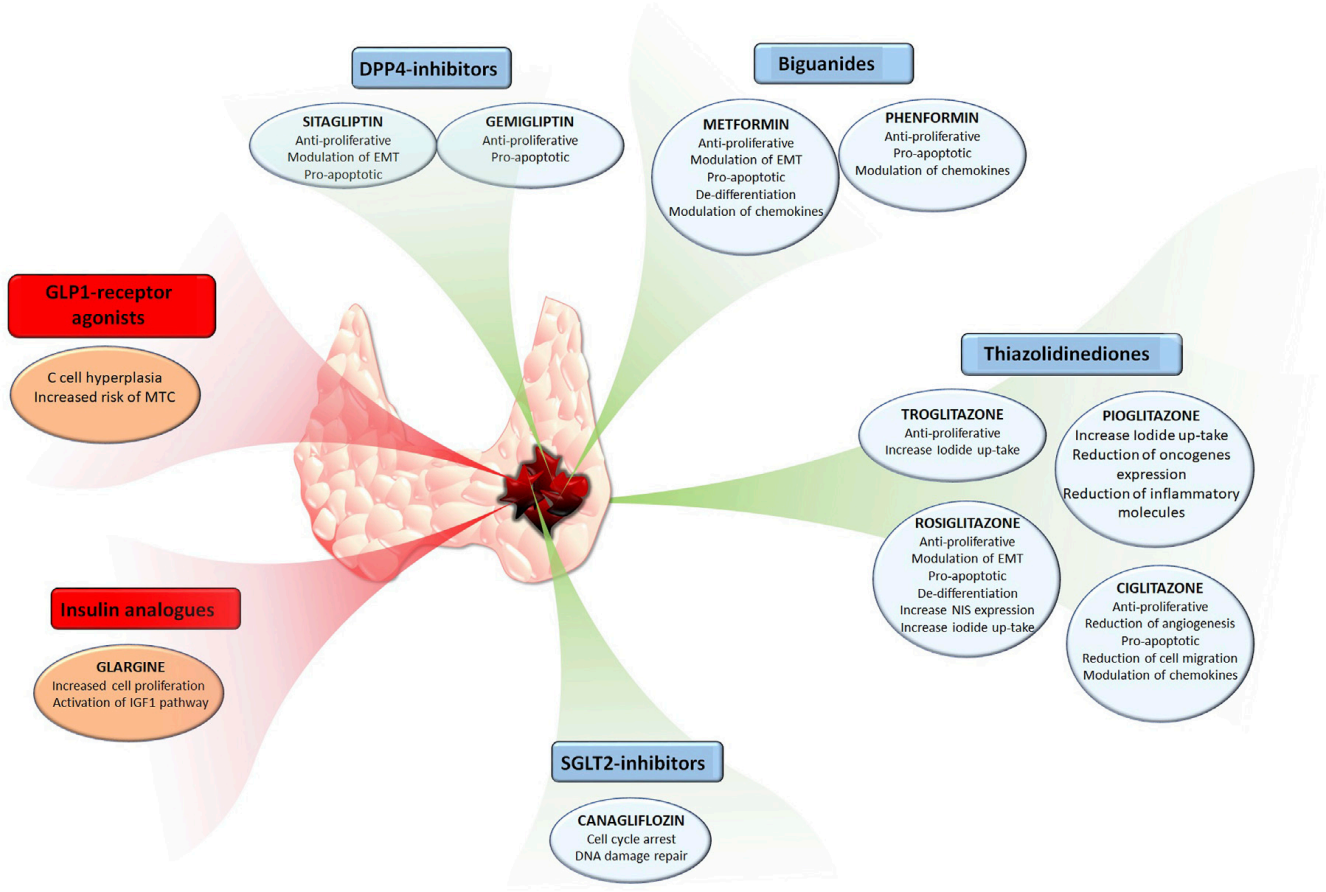
The effects of common anti-diabetic drugs in thyroid cancer.Anti-diabetic compounds exerting anti-cancer effects are have been reported in the blue boxes, also highlighting their respective effects. Anti-diabetic compounds exerting pro-tumorigenic effects are have been reported in the red boxes, also highlighting their respective effects.

The present review encompassed a roundup of studies on the anti-cancer effects of several anti-diabetic compounds in TC. Some of these compounds not only directly affect TC cells by reducing their viability, proliferation, or their ability to migrate to the metastatic side, but also indirectly affect cancer progression by regulating the secretion of pro-tumorigenic chemokines in the TC microenvironment. The reduction of pro-tumorigenic chemokines within and surrounding the thyroid tumor microenvironment is of benefit for counteracting cancer progression. Among the here reported anti-diabetic compounds, it looks like metformin, given its numerous anti-cancer effects which include reduction of cell growth, promotion of cell death, and reduction of cell migration as well as modulation of TC microenvironment component could be of interest for a potential repositioning. Indeed, metformin shows few side effects and encouraging data observed *in vitro* and *in vivo* on TC models. In addition to metformin, other anti-diabetic drugs belonging to other classes, like TZDs and DPP-4 inhibitors showed encouraging results. In this view, it would be of interest to investigate the potential combinatory anti-cancer effects of these compounds. It is important to highlight that not all anti-diabetic drugs appear suitable for repositioning given their potential pro-tumorigenic effects.The effects of anti-diabetica drugs in thyroid cancer are summarized in Figure 1. The example of GLP-1 and medullary TC, although not definitely proven in humans, highlights that anti-diabetic drug repositioning needs to be evaluated specifically for each molecule.

In this view, trials aimed at testing the potential repositioning of these compounds should be designed by also taking into account the mechanism of action of single drugs and potential combination with other drugs or molecules.
